# Rheumatic Hyperpyrexia

**Published:** 1891-08-15

**Authors:** 


### The Practitioner's Mirror and Retrospect.

[*The Editor will be glad to receive offers of co-operation and contributions from members of the profession. All letters should be addressed to* The Editor, The Lodge, Porchester Square, London, W.]

### MEDICINE.

### RHEUMATIC HYPERPYREXIA.

Whatever the true pathological explanation of rheumatic hyperpyrexia may be, there can be little doubt that it exhibits certain clinical features which practically distinguish it from the hyperpyrexia complicating many other diseased conditions. The very rapid occurrence of the hyperpyrexial state, associated with the development of cerebral symptoms, led to the supposition that it was due to inflammatory lesions in the brain or its investing membranes. But post-mortem investigation has shown that such views are erroneous. Sir Thomas Watson and Dr. Latham believed that the cerebral symptoms resulted from cardiac complications, more especially pericarditis.

Fortunately, hyperpyrexia is a rare complication of rheumatic fever, but when it does occur it is often so rapidly fatal that it causes a large percentage of the deaths in the acute stages of rheumatic fever. Dr. Mitchell Bruce states that hyperpyrexia may occur at any period of the disease, generally when the symptoms are fully developed, and even during convalescence; and that the principal indications of the approach of hyperpyrexia, which it is of the last importance to recognise, are flushing of the face, brightness and restlessness of the eyes; an eager, excited expression and behaviour; disappearance of pain and swelling from the joints, and arrest of the perspirations; delirium; and increase of the general symptoms of fever. The decrease in the pain and the brightness of the patient not infrequently mislead the friends if not the medical attendant, and are regarded as happy indications of convalesence. When such symptoms are observed, it is well to be on our guard, and following the advice of Dr. Bruce to take the temperature every half hour. If the body heat prove to be over 103 deg., and to be still rising, measures must be immediately adopted to prevent the hyperpyrexia, which is certainly threatening.

The treatment of rheumatic hyperpyrexia by cold baths was first successfully employed by Dr. Meding, in 1870, and the publication of Dr. Wilson Fox of his treatise on the treatment of hyperpyrexia, with reports of two cases of rheumatic hyperpyrexia successfully treated by cold baths brought the subject prominently before the medical profession of this country. Since then the introduction of many artificially prepared antipyretic drugs, which have proved of such immense service in the treatment of pyrexia, has tended to displace the treatment by cold baths, and consequently the only efficient means of lowering the very high temperature in rheumatic hyperpyrexia is in some danger of being forgotten. According to Hilton Fagge the most probable pathology of this terrible complication is that while the development of the high temperature is itself consequent upon an exhaustion of the heat-regulating centre in the bulb, the cerebral symptoms, in their turn, result from the action of the heated blood upon the brain. The fact that, as a rule, the most active antipyretic drugs fail to effect the reduction of temperature in this condition may be thus accounted for, since the action of such drugs is dependant on the activity of the heat regulating mechanisms.

Dr. Male has published (*Practitioner*, May, 1891) contribution on the treatment of rheumatic hyperpyrexia by cold, which we abstract as follows:—

There are practically but two methods of applying cold to the surface of the body with the view of reducing the temperature; by means of the *bath* and by the *cold pack*.

When the bath is used it is recommended that the patient be lowered into it in a sheet, at a temperature of 90 degs.—100 degs., F.; and that it be cooled down by adding cold water, or preferably pieces of ice, till it is reduced to 60 or 70 degs. He should remain in the bath till the thermometer, placed in the rectum, has fallen to 101 to 102 degs., unless any symptom of faintness or shivering require it to be discontinued earlier. In many cases the patient's temperature continues to fall after his removal from the bath. He must be rubbed dry, and placed in bed lightly covered with blankets; if the temperature fall too low, or the patient is shivering, hot bottles and stimulants are required.

The cold pack is best applied in the following way:—The patient remains in his bed; he is stripped of all clothing and a mackintosh is placed under him. Towels are wrung out of iced water and applied to the trunk, head, and limbs. These are changed frequently, and the body sponged over with lumps of ice. An ice bag should also be applied along the whole length of the spine. The temperature must be carefully watched as before, and the pack discontinued when its reduction has been effected.

Dr. Male thinks that the choice of method to be employed should depend on the circumstances of the case. In hospital practice, where assistance and appliances are at hand, the bath is generally preferred, while in private practice the pack may be preferred for several reasons. Moreover, the latter has proved successful even in extreme cases of hyperpyrexia. Temperatures above 106 degs. F. have been recovered from after treatment by the cold pack, as well as by the bath. In the ten years ending 1890 many cases of recovery have been reported, from time to time, in the journals, and Dr. Male has been able to find a record of sixteen cases with thirteen recoveries; temperatures ranging from 106—110·4 deg. The pack was used in eight of these with two deaths, and the bath in the remaining eight with one death.

While, however, recognising the success that often follows the employment of cold in rheumatic pyrexia, we must ascertain if there are any dangers attending its use. Bristowe records a case whore the cold water on two occasions produced such serious faintness, after five minutes' immersion, that it had to be discontinued. At Guy's Hospital, during 1874 and 1877, death took place on two occasions during immersion; but in these cases the bath was probably too long delayed, and the heart and tissues had suffered too much from the excessive heat to withstand the shock. In several cases violent purgation has resulted after immersion. In a case previously reported by the writer, tetaniform convulsions which occurred after the first application of cold, were so exaggerated on the attempted repetition of the pack that it was impossible to apply it.

These difficulties, however, appear to be exceptional, and it is well to note that the various visceral lesions, such as pericarditis and pneumonia, so common in acute rheumatism, are no contra-indications to the use of cold. Indeed, as Wilson Fox asserts, intense pyrexia predisposes to congestion of internal organs, and the physical signs of such have been frequently observed to clear up during treatment by cold.

